# Swine-Origin Influenza A Outbreak 2009 at Shinshu University, Japan

**DOI:** 10.1186/1471-2458-11-79

**Published:** 2011-02-04

**Authors:** Mitsuo Uchida, Teruomi Tsukahara, Minoru Kaneko, Shinsuke Washizuka, Shigeyuki Kawa

**Affiliations:** 1 Center for Health, Safety and Environmental Management, Shinshu University, 3-1-1 Asahi, Matsumoto 390-8621, Japan

## Abstract

**Background:**

A worldwide outbreak of swine flu H1N1 pandemic influenza occurred in April 2009. To determine the mechanism underlying the spread of infection, we prospectively evaluated a survey implemented at a local university.

**Methods:**

Between August 2009 and March 2010, we surveyed 3 groups of subjects: 2318 children in six schools attached to the Faculty of Education, 11424 university students, and 3344 staff members. Subjects with influenza-like symptoms who were diagnosed with swine flu at hospitals or clinics were defined as swine flu patients and asked to make a report using a standardized form.

**Results:**

After the start of the pandemic, a total of 2002 patients (11.7%) were registered in the survey. These patients included 928 schoolchildren (40.0%), 1016 university students (8.9%), and 58 staff members (1.7%). The incidence in schoolchildren was significantly higher than in the other 2 groups (*P *< 0.0001) but there were no within group differences in incidence rate between males and females. During the period of the survey, three peaks of patient numbers were observed, in November 2009, December 2009, and January 2010. The first peak consisted mainly of schoolchildren, whereas the second and third peaks included many university students. Staff members did not contribute to peak formation. Among the university students, the most common suspected route of transmission was club activity. Interventions, such as closing classes, schools, and clubs, are likely to affect the epidemic curves.

**Conclusion:**

Schoolchildren and university students are vulnerable to swine flu, suggesting that avoidance of close contact, especially among these young people, may be effective way in controlling future severe influenza pandemics, especially at educational institutions.

## Background

A worldwide outbreak of swine-origin influenza A/H1N1 (swine flu) occurred in April 2009 [1-3]. From May 2009 to March 2010, several clusters of swine flu epidemic in Japan caused 15 million infections, resulting in 200 deaths [4]. However, there was no widespread panic among the public during this period, because the Japanese government and many organizations had adopted several prophylactic measures by assuming the worst condition of H5N1 influenza. This pandemic seems now to have passed, but further efforts are necessary to tackle future pandemics of severe influenza infection. In general, systematic and constructive precautions are needed to prevent the expansion of infections among communities. Without adequate preventive measures, catastrophic expansion may occur among densely populated districts. Therefore, the Ministry of Health, Labor, and Welfare of Japan has produced guidelines outlining action plans for new epidemics of influenza infection.

More detailed information, however, is required for systematic and constructive precautions against new outbreaks of these diseases. Correct information concerning the route of infection is especially needed to prevent catastrophic expansion. Surveys in schools or universities may be useful in investigating the mechanisms underlying the spread of infection, because these communities consist of young people who are vulnerable to and at high risk for these epidemics [5-11]. In addition, these communities include older subjects, such as teaching and clerical staff, representing a control population for comparison with data in young people.

We have prospectively evaluated a survey regarding swine flu infection at Shinshu University, a local university in Japan. The study population, which included university students, schoolchildren, and teaching and clerical staff, was monitored throughout the period of the swine flu pandemic. The aim of this study was to identify the risk factors for transmission of swine flu, especially the routes of infection and the manner of infection spread using university members using a descriptive epidemiological method.

## Methods

### Subjects

We prospectively investigated subjects belonging to the Shinshu University Organization from August 2009 to March 2010. The subjects consisted of three groups: 2318 schoolchildren aged 4-18 years old (in Japan, school systems consist of kindergarten (4-6 years old), elementary school (7-12 years old), junior high school (13-15 years old), and high school (16-18 years old)); 11424 university students aged 18-24 years old; and 3344 university staff members, including teaching and clerical staff, aged 22-65 years old. Subjects with influenza-like symptoms and diagnosed with confirmed, probable or suspected swine flu at hospital or clinics, were designated as swine flu patients.

**Figure 1:**
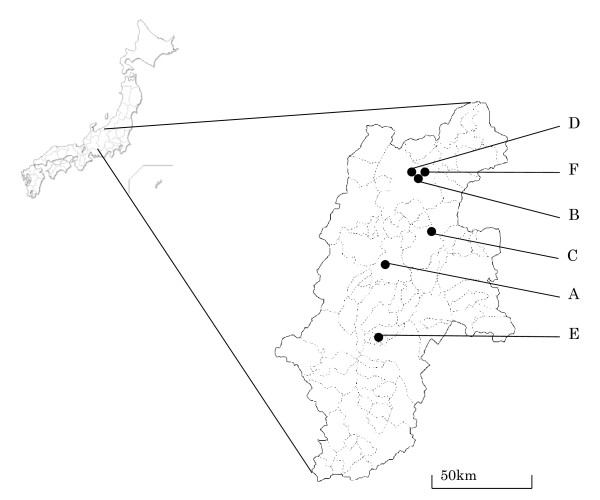
**Location of institutions related to Shinshu University**.

Figure 1 shows the location of each organization in Shinshu University, distributed over six districts (districts A-F) in Nagano prefecture, Japan, at distances ranging from 3 km (B and D) to 120 km (E and F).

Schools attached to the Faculty of Education were located in districts A and F, separated by about 70 km. The schoolchildren in these districts attended 1 kindergarten, 2 elementary schools, 2 junior high schools, and a special education school for mentally or physically handicapped children, all of which were attached to the Shinshu University Faculty of Education. There were 1008 children in district A, including 122 in kindergarten, 422 in elementary school, and 464 in junior high school, and 1310 in district F, including 598 in elementary school, 657 in junior high school, and 55 in special education school. Of the university students, 2301 freshmen had been admitted to the university in 2009 and were in the School of General Education in District A, taking the same general education classes. There were also 9123 upper classmen belonging attending various schools in five districts. In district A, there were 865 students in the Faculty of Economics, 601 in the Faculty of Arts, 933 in the Faculty of Science, and 1136 in the School of Medicine. In district B there were 2417 students in the Faculty of Engineering; in district C, 1417 students were in the Faculty of Textile Science and Technology; in district D, 1005 students were in the Faculty of Education; and in district E, 749 students were in the Faculty of Agriculture. Of the university staff, 2443 belonged to organizations in district A, while the others, numbering 106 to 284, belonged to the organizations of the other 6 districts.

### Procedure

To prevent pandemic infection, a systematic survey system for swine flu patients was established, with all information collected at university headquarters and the Department of Health, Safety, and Environmental Management. Patients were instructed to telephone the designated person in each organization. Telephone inquiries were made for each patient using a standardized case report form that included the date of onset, initial symptoms, diagnosis by clinician, suspected infection route, and prescription medicine. Collected records were faxed immediately to the university headquarters. During the telephone call, the designated person also suggested that the patient should not go to school until 2 days after his or her body temperature returned to normal. Information on infection was statistically adjusted based on collected records and disclosed daily on the Shinshu University web site. All infected university students and staff members were asked to report the details of infection by telephone; however, information on infected schoolchildren was collected only by their teachers. Therefore, the records for schoolchildren with influenza-like symptoms were less complete than the records for university students and staff.

### Precautions and Infection Control

All subjects were encouraged to practice precautions, such as gargling, washing hands, and wearing a mask, through a circular, university website, and/or cellular phone text messages. Patients with influenza-like symptoms were directed to stay home until 2 days after their body temperature had returned to normal, with this period considered to be an authorized absence. Temporary school or class closure was performed when the number of infected patients reached the prescribed proportion of 10% in a given week or was decided by the principal of each school. Club activities for university students were prohibited for one week when more than 2 members were infected at the same time.

Vaccination for swine flu in Japan began in October 2009. The government gave priority for vaccination to specific high-risk groups, including medical staff, pregnant women, patients with specific diseases, and children aged approximately 1 to 10 years old, to prevent severe illness. Therefore, few university students or staff members could have been vaccinated for swine flu during the study period. In addition, as the vaccine was not available in sufficient amounts in the districts examined during the study period, few schoolchildren would have been vaccinated.

### Statistical analysis

We used a descriptive epidemiological method for this research. The number of patients was counted every week, with each week considered a unit. For categorical variables, the percentages of patients in each category were calculated and the proportions were compared using the χ^2 ^test. In all analyses, *P *< 0.05 was considered statistically significant. All analyses were performed using PASW 18 software for Windows.

### Ethics

We essentially analyzed the records of swine flu patients. Although the records included individual information at the time of data collection, the data were de-identified at the time of analysis. As 2009 swine flu is a nationally notifiable disease in Japan and this epidemiological study would not cause any disadvantages to any of the subjects, written informed consent from each patient was not required. The study design and procedure were reviewed and approved by the Committee for Medical Ethics of Shinshu University (approval number 1616).

## Results

### Demographic characteristics and clinical symptoms of the patients

The demographic characteristics of each group are shown in Table 1, along with information on numbers of patients and infection rates for both sexes. The total number of patients was 2002 (11.7% of all subjects). All patients were reported once in the database during the study period, with multiple reports from individuals not shown. Of these 2002 patients, 928 were schoolchildren (40.0% of all children), 1016 were university students (8.9% of all students), and 58 were staff members (1.7% of all staff members), with the infection rate among schoolchildren being significantly higher than in the other 2 groups (*P *< 0.0001). Within each subgroup, the infection rate did not differ significantly between males and females ("Male%" indicates the infection rate among males in each group). The majority of infected individuals had mild illness, with only four patients requiring hospitalization (2 schoolchildren and 2 university students, all of whom had respiratory illness). None of the subjects in this study died.

**Table 1:** Numbers of patients and infection rates among schoolchildren, university students, and staff.

Group	District	Subgroup	Subjects	Patients	%	Male%		Female%
**Schoolchildren**								
	A	Kindergarten	122	43	35.2	39.7	/	31.3
		Elementary school	422	228	54.0	54.5	/	53.6
		Junior high school	464	212	45.7	50.5	/	41.4
	F	Elementary school	598	202	33.8	34.3	/	33.3
		Junior high school	657	228	34.7	34.0	/	35.4
		Special education school	55	8	14.5	8.8	/	23.8
		Incomplete		7				
		Total (Average)	2318	928	(40.0)*	(40.5	/	39.0)
**University students**								
	A	Freshmen	2301	357	15.5	17.3	/	14.1
		Arts	601	28	4.7	5.5	/	4.2
		Economics	865	49	5.7	4.7	/	7.0
		Science	933	51	5.5	5.2	/	6.1
		School of Medicine	1136	70	6.2	6.4	/	4.6
	B	Engineering	2417	105	4.3	4.8	/	2.0
	C	Textile Science and Technology	1417	149	10.5	9.7	/	13.1
	D	Education	1005	101	10.0	10.2	/	9.9
	E	Agriculture	749	93	12.4	13.2	/	12.5
		Incomplete		13				
		Total (Average)	11424	1016	(8.9)*	(8.7	/	9.2)
**Staff**								
	A		2443	30	1.2	1.3	/	1.1
	B		284	1	0.4	0.4	/	0.0
	C		233	10	4.3	2.0	/	7.0
	D		143	3	2.1	3.0	/	0.0
	E		135	5	3.7	3.9	/	6.5
	F		106	9	8.5	7.2	/	11.4
		Total (Average)	3344	58	(1.7)*	(1.6	/	1.7)

**All subjects**		Total (Average)	17086	2002	(11.7)	(10.9	/	12.5)

* χ^2 ^test (*P *< 0.0001)

In addition to fever, the complaints and symptoms in infected university students and staff included cough (71.9%), headache (41.7%), runny nose (37.1%), sore throat (33.3%), arthralgia (30.6%), nausea (5.8%), diarrhea (3.7%), abdominal pain (2.5%), and vomiting (0.8%). Thus, infection was accompanied by major respiratory symptoms and minor gastrointestinal symptoms. All of these patients were prescribed one or more medicines for their complaints and symptoms (oseltamivir, zanamivir, or NSAIDS) at hospitals and clinics.

### Serial changes in patient number from August 2009 to March 2010

**Figure 2:**
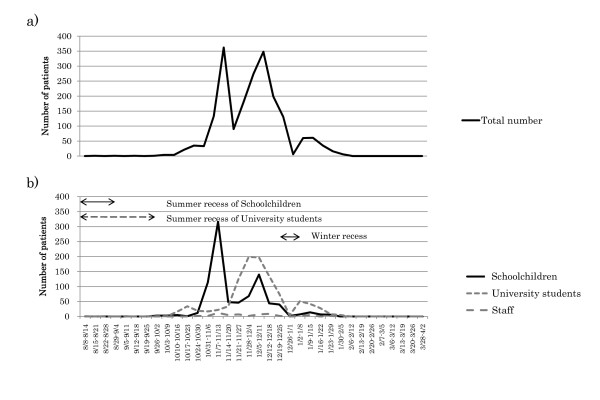
**Serial changes in patient number from August 2009 to March 2010**. a) Total numbers of patients per week of the study period. b) Serial changes in patient numbers for the 3 groups. Attached schools had summer recess in August, with school classes resuming in September 2009, whereas university classes had recess in August and September and restarted in October 2009. All subjects had winter recess from the end of December 2009 to the beginning of January 2010.

Figures 2a and 2b show the total number of patients and the numbers of patients in each of the 3 groups per week during the study period. All schoolchildren had summer recess in August 2009, with classes resuming in September 2009. In contrast, university students had recess in August and September 2009 and restarted in October 2009. Moreover, both schoolchildren and university students had winter recess from the end of December 2009 to the beginning of January 2010. In Japan, the first case of swine flu was identified in May 2009, and the epidemic spread around August 2009. However, it was delayed in this area, with infected patients observed starting in October 2009, or after summer recess. Patient numbers peaked in November 2009, December 2009, and January 2010, with the latter peak seemingly influenced by winter recess.

**Figure 3:**
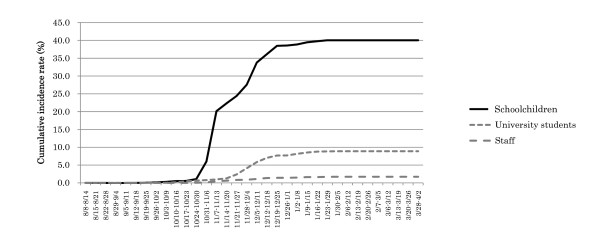
**Cumulative incidence rates of patients in the 3 groups**.

Serial changes in patient numbers for the 3 groups--schoolchildren, university students, and staff members--indicated that the first peak began slowly in university students and consisted mainly of schoolchildren, the second peak consisted of both schoolchildren and university students, and the third peak consisted primarily of university students, in contrast, staff members did not contribute to formation of any of these peaks (Figure 2b). The cumulative incidence rates of patients were 40.0% for schoolchildren, 8.9% for university students, and 1.7% for staff members (Figure 3).

### Comparison of schoolchildren patients in districts A and F

**Figure 4:**
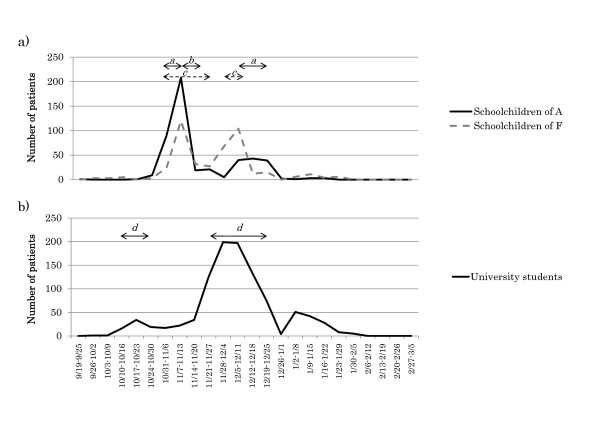
**Epidemic curves and interventions**. a) Epidemic curves and interventions of attached schools in districts A and F. Arrows indicates the periods of class closures (*a *and *c*) and a school closure (*b*). Each class closure ranged from 1 to 10 days, with some classes closed simultaneously. Kindergartens, elementary schools, and junior high schools in district A were closed simultaneously for 4 days, but only once. b) Epidemic curve and interventions of club closures (*d*) among university students. Club activity was stopped when more than two attendees were infected at the same time. Some clubs were closed simultaneously in October and November 2009, and the number of patients decreased after the interventions.

Serial changes in numbers of schoolchildren patients were observed in districts A and F (Figure 4a). Although these two districts are located 70 km from each other, the epidemic in schoolchildren occurred simultaneously, showing 2 identical peaks in November and December 2009. Schoolchildren in these districts were encouraged to practice precautions, such as gargling, washing hands, and wearing masks. Schools or classes in these two districts were closed temporarily but at different times. The cumulative numbers of patients (cumulative incidence rate) in districts A and F were 485 (48.1%) and 443 (33.8%), respectively.

### Patient numbers among university students

We found that the infection rate among university students in each district ranged from 4.3% to 15.5% (Table 1). The infection rate was significantly higher among freshmen than among upperclassmen (15.5% *vs. *7.0%; *P *< 0.0001). In addition, collected records indicated that the most common suspected route of infection among university students was club activity (29.8% in all students and 35.1% in freshmen). Club activity was stopped when more than two members in that club were infected at the same time, and this closure was likely to affect the epidemic curve in university students (Figure 4b).

## Discussion

We have described here the epidemiology of an outbreak of swine flu between August 2009 and March 2010 at Shinshu University, Japan. Although 2002 patients were infected during this period, only a few developed severe conditions and were hospitalized but none died. Major complaints were respiratory symptoms, similar to seasonal influenza [9,12]. However, this swine flu is known to cause severe acute distress in some patients with chronic diseases, such as obesity, diabetes, and/or respiratory disease [13-15]. Therefore, intensive systemic precautions and infection control will be required to combat upcoming influenza pandemics.

The infection started slowly in university students and spread mainly through club activities, indicating that close contact with sick individuals is a major factor for transmission. The epidemic subsequently occurred in schoolchildren, suggesting that infection may have spread through family members or other routes. We could not confirm close relationships between university students and schoolchildren, through which the infection could have spread. In addition, the present swine flu epidemic in Shinshu University formed 3 peaks of infected subjects, consisting primarily of schoolchildren and university students. The first peak in November 2009 consisted mainly of schoolchildren, the second peak in December 2009 consisted of university students and schoolchildren, and the third peak in January 2010 consisted mainly of university students. The first peak seemed to be affected by summer recess, in that the pandemic was recognized in August 2009 in Japan. Moreover, winter recess likely affected the epidemic curves for both schoolchildren and university students simultaneously. Gradual decreases in all 3 peaks seemed to be achieved by implementation of precautions and infection control measures, such as temporary school and class closings and the prohibition of meetings and club activities. Our findings are in good agreement with previous reports, which indicated that school closures during the H1N1 pandemic was a very effective means of reducing the number of infected individuals [5,16,17]. Moreover, our findings also indicate that controlled inhibition of close contact among members of an organization is an effective way to prevent the transmission of swine flu as well as seasonal influenza.

We found that the infection rates differed significantly among our 3 groups, schoolchildren, university students, and staff members. Previous studies have indicated that swine flu infection is more prevalent in younger than in older people [5,9-12]. We found that the infection rate in schoolchildren was highest among the three groups. In addition, the cumulative incidence rates among children reached a plateau of 40.0% (average), in agreement with previous reports indicating that the incidence of disease can be effectively reduced or prevented if 30%-60% of individuals at school could be vaccinated [18]. However, the infection rates among university students and staff were only 8.9% and 1.7%, respectively. This disparity seemed to be due to differences between younger and older people in their understanding the importance of precautions, skill in taking precautions, and practice of precautions. These results thus provide some additional evidence for the effective control of H1N1 influenza.

The first epidemic in schoolchildren occurred simultaneously in districts A and F, although these 2 districts were located about 70 km from each other. These results indicated that pandemic outbreaks may first occur in young children who cannot practice sufficient precautions, such as avoidance of close contact, thereafter spreading over a broad area beyond the city or prefecture. Transmission through household contact with siblings may also contribute to the spread of infection among this group. The susceptibility to infection with the 2009 H1N1 virus from a household member has been estimated to be twice as high in children as in adults 19 to 50 years old, with adults older than 50 years being less susceptible than younger adults [16]. Further studies are required to determine the geographical effects on pandemic transmission, especially in young children.

Among university students, freshmen had a significantly higher incidence rate than students in other grades. Among the reasons for this higher rate in freshmen were: 1) all freshmen were gathered in one district, where they were in relatively close contact with each other during club activities, lectures, and side jobs; and 2) freshmen maintained close contact with each other because they were required to take part in group activities. Among these factors, close contact associated with club activity may have played a major role in the spread of infection. In contrast, upper grade university students were separated into various districts, where they had little chance of participating in group activities. Consequently, among university students, H1N1 transmission may have been easier in freshmen than in upper classmen. Our findings indicate that different types of precautions and infection control measures are required in university settings, depending on the characteristics of each group, *i.e.*, schoolchildren, freshmen students, upper grade students, and staff.

Our study had several limitations. First, statistical analysis was based on records of patients, including those with confirmed, probable or suspected swine flu. In general, infection with swine flu virus requires confirmation by real-time reverse transcriptase polymerase chain reaction (RT-PCR) or viral culture, according to the CDC and WHO definitions, but these laboratory tests were not performed for all of our patients. Therefore, our patient population may have included individuals infected with seasonal influenza virus, and we therefore may have overestimated the number of patients with swine flu infections. However, according to the Ministry of Health, Labor, and Welfare and the National Institute of Infectious Diseases of Japan, swine flu accounted for almost all (99.2%) infections with influenza virus during this period (http://idsc.nih.go.jp/disease/swine_influenza/webcast/pdf/3.pdf). Therefore the patients from our organization during the study period were regarded as having swine flu. Second, we could not collect detailed information for schoolchildren, including their symptoms, medicine prescribed, or suspected route of infection. Therefore, this information could not be analyzed for all subjects. Since infection was spread in the school, the designated individuals in each district could not complete telephone reports by speaking with the parents of these schoolchildren. Therefore, we could only partly evaluate the symptoms of swine flu among these patients.

## Conclusions

Shinshu University, Japan, experienced a pandemic of swine flu from August 2009 to March 2010. Few patients were hospitalized and there were no deaths, because several precautions and infection control measures were effectively carried out.

Despite some limitations, this study provided additional evidence regarding measures that can be taken within an organization to limit the spread of swine flu. The strengths of this study are related to the collection of the records of all infected patients in a large organization during an epidemic. The records covered all patients among schoolchildren, university students, and university staff members. Our results suggested that avoidance of close contact, especially among younger people, is the most important way to control an influenza pandemic. In future, further interventions and improved quarantining of infected patients during outbreaks of highly toxic influenza, such as pandemic H5N1, would be useful.

## Competing interests

The authors declare that they have no competing interests.

## Authors' contributions

MU designed and oversaw the study, performed the statistical analysis and wrote the manuscript. TT, MK, SW and SK proposed suggestions to improve the study and revised the manuscript. All authors read and approved the final manuscript.

## Pre-publication history

The pre-publication history for this paper can be accessed here:

http://www.biomedcentral.com/1471-2458/11/79/prepub
